# A demonstration of modeling count data with an application to physical activity

**DOI:** 10.1186/1742-5573-3-3

**Published:** 2006-03-21

**Authors:** Donald J Slymen, Guadalupe X Ayala, Elva M Arredondo, John P Elder

**Affiliations:** 1 Graduate School of Public Health, San Diego State University, 5500 Campanile Dr., San Diego, CA 92182, USA; 2 San Diego State University, Center for Behavioral and Community Health Studies, 9245 Sky Park Ct., Suite 221, San Diego, CA 92123, USA

## Abstract

Counting outcomes such as days of physical activity or servings of fruits and vegetables often have distributions that are highly skewed toward the right with a preponderance of zeros, posing analytical challenges. This paper demonstrates how such outcomes may be analyzed with several modifications to Poisson regression.

Five regression models 1) Poisson, 2) overdispersed Poisson, 3) negative binomial, 4) zero-inflated Poisson (ZIP), and 5) zero-inflated negative binomial (ZINB) are fitted to data assessing predictors of vigorous physical activity (VPA) among Latina women. The models are described, and analytical and graphical approaches are discussed to aid in model selection.

Poisson regression provided a poor fit where 82% of the subjects reported no days of VPA. The fit improved considerably with the negative binomial and ZIP models. There was little difference in fit between the ZIP and ZINB models. Overall, the ZIP model fit best. No days of VPA were associated with poorer self-reported health and less assimilation to Anglo culture, and marginally associated with increasing BMI. The intensity portion of the model suggested that increasing days of VPA were associated with more education, and marginally associated with increasing age. These underutilized models provide useful approaches for handling counting outcomes.

## Introduction

Counting outcomes are often characterized by a large proportion of values at zero with the remaining values highly skewed toward the right. This type of distribution may occur in physical activity measures in which number of days of physical activity may be of interest, nutritional measures assessing, for example, the number of servings of fruits and vegetables, or health services describing the number of doctor or hospital visits. Since counting outcomes do not meet the usual normality assumption required of many standard statistical tests, analysts have relied on a transformation to induce normality, which often does not work, or categorization of the outcome which may result in loss of information. An alternative is to assume a Poisson distribution which is better suited to counting processes. Software for Poisson regression is readily available and this technique is becoming more widely used in many fields of research. However, a necessary condition of the Poisson distribution is that the expected value is equal to the variance. Many counting outcomes exhibit more variability than the nominal variance under the Poisson model, a condition called over-dispersion. The consequences can be severe if over-dispersion is not addressed. Confidence intervals for regression estimates may be too narrow and tests of association may yield p-values that are too small. Modifications to the Poisson model have been proposed both to account for over-dispersion and to understand better the underlying process which leads to such a highly skewed distribution. These techniques have been used in the social science literature [1,2] and, to some extent, in health services research [e.g. [3]]. However, few examples are available in public health and clinical research. Some recent examples include a study to examine early child growth and development [4], the use of male condoms in a behavioral intervention study [5], an epidemiologic study of risk factors for severe hypoglycemia where the outcome was number of episodes over a 9-year period [6], modeling the relationship between number of dental caries and socioeconomic status [7], and a series of articles on motor vehicle crashes [8-10]. A closely related problem is continuous outcomes with a high proportion (or clumping) of zeros [11,12]. Chang and Pocock [13] describe two approaches for analyzing such data based on 1) categorizing the outcome and fitting a proportional odds model or 2) modeling the probability of a zero response using logistic regression and then least squares linear regression for the non-zero part. Modifications to the Poisson distribution have also been proposed in other applications, for example, estimating confidence intervals for incidence rates where the case count may be inflated due to false positives [14]. In the current paper, the outcome is a counting process where linear regression would not be appropriate and where the conceptual framework concerning the preponderance of zeros is explored. We will provide examples of modifications to the Poisson and apply them to a study assessing predictors of vigorous physical activity (VPA) at baseline among Latina women participating in a randomized nutrition intervention trial.

## Analysis

### Physical activity example

The data presented in this example were gathered as part of a baseline measurement from a randomized community trial. The NIH-funded trial, *Secretos de la Buena Vida*, examines the effectiveness of two innovative communication approaches to improving the nutritional health of adult Latinas/Hispanics by contrasting these participants to those in a "usual care" control group. Adult women were recruited via random digit dialing from the Central and Southern regions of San Diego County based on a list of phone numbers purchased according to region using zip codes and with a Hispanic surname on the account. To be eligible for the study, the household must have included a woman whose dominant language was Spanish and was between 18 and 65 (inclusive) years of age.

The data were collected during face-to-face home-based interviews with 357 female participants. Women who agreed to participate were measured at baseline and then randomly assigned to one of three conditions: promotora plus tailored print material delivered via mail condition (*promotora*); mailed tailored print only (tailored); and a control condition (control; off the shelf Latino targeted materials, also delivered via mail). Women in the *promotora *condition received weekly home visits or telephone calls from *promotoras *(lay health advisors in the Latino community) over a 12-week period plus 12 weekly tailored newsletters with activity inserts mailed to the participant's home. The tailored condition received tailored newsletters and activity inserts created especially for the woman using information provided by the participant at baseline. The control group was mailed "off the shelf" materials covering the same content areas as those in the *promotora *and tailored conditions.

The primary outcome variables for this study were percent calories from fat and number of grams of fiber. The Nutrition Data System (NDS) 24-hour dietary recall interview, developed by the Nutrition Coordinating Center (NCC) of the University of Minnesota, was used at each measurement to assess dietary consumption patterns. This longitudinal research study included 4 repeated assessments over an 18-month period. Details of the study design and procedure may be found elsewhere [15].

As part of the baseline questionnaire several questions were asked concerning physical activity and regular exercise. In particular, the following information and question were presented:

"Vigorous physical activities usually make you breathe hard or feel tired most of the time. Examples of vigorous activities include: jogging, fast dancing, soccer, fast swimming, fast biking, and Stairmaster. How many days in a typical week do you do vigorous physical activities for 20 minutes or more?"

A literature search [16-26] was carried out to determine appropriate predictors of physical activity for this demonstration. The selected variables are shown in Table 1. Pertaining to body mass index, weight was measured three times to the nearest pound using a Health-o-Meter standard scale and height was measured three times to the nearest 1/4 inch, using a standard portable stadiometer with shoes removed. Mean weight and height scores were computed based on the three measurements. BMI was calculated using the Quetelet index (kg/m^2^), which is considered both a convenient and reliable indicator of overweight and obesity [27].

**Table 1:** Descriptive Statistics for Number of days of VPA and Predictor Variables

Variable	Frequency (%) or Mean (SD)
Number of days/week of VPA of 20 minutes or more	
0	294 (82.4)
1	10 (2.8)
2	12 (3.4)
3	17 (4.8)
4	7 (2.0)
5	10 (2.8)
6	0 (0.0)
7	7 (2.0)
	
Current employment status	
Full-time	91 (25.5)
Part-time	50 (14.0)
Self-employed	33 (9.2)
Homemaker/other unemployed	183 (51.3)
	
Years of formal education	
Through 6^th ^grade	95 (26.6)
Middle school	89 (24.9)
High school	76 (21.3)
Any college	97 (27.2)
	
Marital status	
Married	280 (79.1)
Not married	74 (20.9)
	
Smoked cigarettes past 30 days	
Yes	52 (14.6)
No	304 (85.4)
	
Self-reported health	
Excellent (1)	20 (5.6)
Very good (2)	34 (9.6)
Good (3)	103 (28.9)
Fair (4)	183 (51.4)
Poor (5)	16 (4.5)
	
Numerical score mean (sd)	3.4 (0.93)
	
Body Mass Index	29.6 (5.56)
Household size	4.7 (1.78)
Age (years)	39.7 (9.93)
Acculturation score	-1.82 (0.90)

Acculturation was measured using a score derived from the 30-item Acculturation Rating Scale for Mexican-Americans-II (ARSMA-II) [28]. This scale measures frequency of using the English and Spanish languages, frequency of accessing English and Spanish media (TV, movies, music, books, newspapers), frequency of interacting with Mexicans and Anglos now and as a child, and ethnic identity of self and parents. Separate scores are obtained representing 1) Mexican orientation or the frequency with which she speaks Spanish, watched Spanish-language television, and identifies as a Mexican and 2) Anglo orientation or the frequency with which she spoke English, watched English-language television and identified as an American or Mexican-American. The acculturation score is obtained as the difference between the two scores (Anglo minus Mexican orientation). Thus, a negative score represents a Mexican orientation and a positive score an Anglo orientation.

Although the literature also identified income as a predictor of physical activity, we found that it was very highly associated with education in our sample and income level was missing on 19 subjects. Therefore, we decided to exclude income and use education status.

### Statistical models

Let y_i _represent the count for the i^th ^subject. Let **x**_i _be a vector of covariates and ***β*** a vector of regression coefficients to be estimated. The Poisson regression model may be represented as:



where *μ*_i _= exp(**x**_i_' ***β***). The expected value of y_i _given **x**_i _is *μ*_i_. The variance of y_i _is also *μ*_i_.

Although Poisson regression is often used for counting outcomes, the observed counts often exhibit more variability than what is predicted by the Poisson, a condition called over-dispersion. This leads to underestimation of the standard errors of the regression estimates, confidence intervals that are too narrow, and p-values that are too small. In some cases, the underestimation can be very severe. The extra variation can be measured by a dispersion or scale parameter. The parameter may be estimated by dividing a goodness-of-fit statistic by the residual degrees of freedom [29]. The parameter estimate is routinely included in the output of computer software for Poisson regression [e.g. [30], p. 360]. If the estimate is greater than 1, there is evidence of over-dispersion. The estimate may then be used as a scaling factor, multiplying the estimated covariance matrix of ***β*** by this quantity. This serves to "inflate" the standard errors with confidence intervals wider and p-values larger than what is obtained under the Poisson without adjustment for over-dispersion.

Another approach to managing over-dispersion within the framework of Poisson regression is to use a generalized estimating equation (GEE) approach [[30], pp 542–7]. Over-dispersion may be thought of as misspecification of the covariance structure, one that is not appropriate for Poisson regression. Rather than use the model-based covariance estimate, GEE uses a robust estimate that is based on a subject-to-subject measure for estimating variances. Stokes et al provide an example of its use.

Gardner et al. [29] suggest that using an inflation technique to handle over-dispersion may be adequate if the intent of the analysis is to test hypotheses about the regression coefficients. If one of the objectives is to estimate probabilities for individual counts, then alternative models should be explored.

It may be worthwhile to consider the mechanism by which the over-dispersion occurs and use a more flexible regression model. Even after taking into account the covariate information, there may be unexplained variability among subjects, possibly a result of unobserved predictors. The mean *μ*_i _may be replaced by

*μ*_i_* = exp(**x**_i_' ***β***+ *ε*_i_) where *ε*_i _represents random error. Now, subjects with the same observed **x**_i _do not share the same *μ*_i _due to the unobserved heterogeneity. This modification to the Poisson regression yields a more flexible regression model, the negative binomial:



where *θ *represents the degree of over-dispersion. The mean is *μ*_i_, the same as the Poisson, but the variance is *μ*_i_(1 + *θμ*_i_) thus allowing the variance to exceed *μ*_i _. As *θ *approaches 0, the negative binomial approaches the Poisson. If both models are fitted, a likelihood ratio test may be used to compare them. Alternatively, score tests have been developed which require only fitting the Poisson regression model [31,32].

Another approach is to consider the excess of zeros by hypothesizing that there are two groups contributing to the amount. Within the context of vigorous physical activity, there is a subpopulation of subjects who never engage in any vigorous physical activity and are not contemplating any change in behavior. The second group reports no vigorous physical activity but are more amenable and have the potential to increase their physical activity; they simply have not yet reached a reporting threshold beyond zero. Thus, they are part of a Poisson process which includes a portion of zeros but models an increasing involvement in vigorous physical activity. The first group is not part of this process.

Lambert [33] proposed a zero-inflated Poisson (ZIP) model:



where p_i _is the probability of being an extra zero. In the ZIP model p_i _is determined, typically, by either a logistic or a probit model, g(**z**_i_'**γ**). And *μ*_i _is again modeled as exp(**x**_i_'***β***). The x's and z's may represent the same set of covariates or be different sets. Substituting the negative binomial for the Poisson yields the zero-inflated negative binomial (ZINB) model.

This study examines and compares the Poisson, over-dispersed Poisson, negative binomial, ZIP and ZINB regression models.

### Model comparisons

As mentioned earlier, a likelihood ratio test may be constructed to compare the Poisson to the negative binomial since the Poisson is nested within the negative binomial. This is a test of *θ *= 0 and follows a chi-square distribution with 1 degree of freedom. Similarly, the ZIP model is nested within the ZINB. Comparisons of the Poisson with the ZIP and the negative binomial with the ZINB involve nonnested comparisons. A common approach to model selection in such instances is to use the Akaike Information Criterion (AIC) where the model chosen minimizes the criterion [34]. The AIC is available in many computer packages. In addition we plot the observed minus predicted probabilities from each model to obtain graphical illustrations of fit. The predicted probabilities are constructed using the approach in Long [[1], p. 228] and are adjusted for all covariates.

All models are fitted using SAS version 8.1 [35]. The Poisson, over-dispersed Poisson, and negative binomial are fitted using the GENMOD procedure. The ZIP and ZINB models are fitted with the NLMIXED procedure. However, packages such as STATA [36] and LIMDEP [37] also have specific programs for fitting such models.

## Results

Table 1 displays the frequency distribution for number of days of VPA. Of the 357 women, 294 (82.4%) report no days of VPA. For days 1 to 3 the percentages range from 2.8% to 4.8%, dropping off to 0% to 2.8% for days 4 to 7. Although it may be convenient to simply lump days 1 to 7 together and dichotomize the outcome as 'none versus any VPA, there is useful information in retaining the intensity portion of the response. Clearly, a transformation to induce normality with such positively skewed data is not feasible.

The selected predictor variables are also shown in Table 1. For current employment status, over 50% are classified as "homemaker/other unemployed". However, all but 13 of the 183 responses represent "homemaker". The remainder consists of "student" (2), "retired" (1) and "unable to work" (10), all reflecting non-employment categories. Years of formal education are evenly distributed across the four categories. The sample is predominantly married (79.1%), does not engage in smoking on a regular basis (85.4%) and has fair to good self-reported health (80.3%). For purposes of analysis, self-reported health is treated as a continuous variable. The mean age is nearly 40 years old. The mean body mass index (29.6 kg/m^2^) suggests the sample is borderline obese (BMI ≥ 30.0 kg/m^2^). The mean acculturation score of -1.82 indicates the sample tends to be oriented toward the Mexican culture.

Poisson regression was fitted to the data using the GENMOD procedure of SAS. The ratio of the deviance to its degrees of freedom (df) is 1.99 and the ratio of the Pearson chi-square to df is 3.27, both indicating over-dispersion. The negative binomial regression model was fitted and yielded a log-likelihood of -282.8. The likelihood ratio test comparing the negative binomial to the Poisson, which tests H_0_: *θ *= 0, yields a statistic of 277.4. The estimate of *θ *is 6.94 (SE = 1.33). The negative binomial is favored over the Poisson. Table 2 displays the regression and standard error estimates and p-values for the Poisson, over-dispersed corrected Poisson using GEE and the negative binomial regression. The over-dispersion adjustment affects only the standard errors, not the regression estimates. Note that the Poisson standard errors tend to be nearly half the size of the GEE-corrected standard errors leading to considerably (and incorrect) smaller p-values. The negative binomial compared to the Poisson affects both the regression and standard error estimates. Although the GEE-corrected results and negative binomial produce somewhat different regression estimates, both identify BMI, self-reported health and acculturation score as associated with VPA (p < 0.05).

**Table 2:** Poisson, Over-dispersed Poisson using GEE, and Negative Binomial Models for Number of Days of VPA

				Over-dispersed				
	Poisson Regression			Poisson		Negative Bionomial		
	Beta	SE	p-value	SE	p-value	Beta	SE	P-value
Current employment status								
Full-time	Reference			Reference		Reference		
Part-time	0.474	0.235	0.044	0.419	0.26	0.274	0.569	0.63
Self-employed	0.595	0.274	0.030	0.512	0.25	0.177	0.716	0.81
Homemaker/other unemployed	0.493	0.201	0.014	0.405	0.22	0.434	0.450	0.33
								
Years of formal education								
Through 6^th ^grade	Reference			Reference		Reference		
Middle school	-0.006	0.249	0.98	0.431	0.99	0.356	0.527	0.50
High school	0.349	0.237	0.14	0.471	0.46	0.236	0.536	0.66
Any college	0.369	0.231	0.11	0.445	0.41	-0.041	0.605	0.95
Smoked cigarettes past 30 days (Y/N)	-0.398	0.217	0.066	0.361	0.27	-0.884	0.552	0.11
Body Mass Index	-0.069	0.016	<0.0001	0.025	0.006	-0.094	0.042	0.025
Marital status (Married/Not married)	-0.194	0.212	0.36	0.423	0.65	-0.416	0.477	0.38
Self-reported health (1=Excellent, 5=Poor)	-0.357	0.072	<0.0001	0.123	0.004	-0.572	0.233	0.014
Household size	0.001	0.046	0.99	0.108	0.99	-0.044	0.089	0.63
Age (5 yr interveal)	-0.019	0.040	0.64	0.089	0.84	-0.083	0.090	0.36
Acculturation score	0.267	0.086	0.002	0.128	0.037	0.556	0.271	0.040

**Figure 1:**
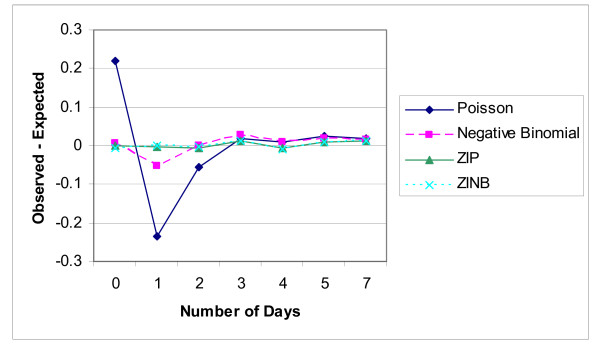
Observed minus expected probabilities for four models.

Next, the ZIP and ZINB models were fitted using the NLMIXED procedure of SAS. There is virtually no difference in their log-likelihoods (Table 3) indicating that the ZINB model did not improve the fit over the ZIP model. The over-dispersion parameter estimate is 0.0062 (SE = 0.106). As expected based on these results, the parameter estimates and standard errors of the estimates are nearly identical. The AIC's in Table 3 indicate a strong preference of the ZIP over the Poisson model and the ZINB over the negative binomial model. Overall, the AIC criterion favors the ZIP model. Finally, Figure 1 plots the observed proportion minus the mean probability at each count for each of the four models. It is clear that the Poisson provides the worst fit. At 0 days the observed proportion is 0.2 higher than the expected; at 1 day, the reverse occurs. This is not surprising since the Poisson is unable to account for the large proportion of zeros. While the negative binomial is a substantial improvement over the Poisson, at 1 day there is some overestimation of the proportion. The ZIP and ZINB models are virtually indistinguishable on the plot and both fit the data quite well. Based on the formal tests and the figure, the ZIP model would appear to be the best fit. It provides the same fit as the ZINB, but is a somewhat simpler model and has a slightly smaller AIC.

**Table 3:** Model Fit Characteristics: Log-likelihood and Akaike's Information Criterion

Model	Log-likelihood	AIC
Poisson	- 421.5	871.1
Negative binomial	- 282.8	595.6
Zero-inflated Poisson	- 253.3	562.5
Zero-inflated negative binomial	- 253.5	565.0

Table 4 displays the results for the ZIP model. The odds ratios and rate ratios are reported rather than the regression estimates consistent with how information from logistic regression and Poisson regression are typically reported. The logistic portion is based on the probability of zero days of vigorous physical activity. Poorer self-reported health is related (p = 0.023) to no vigorous physical activity. The odds of no VPA are estimated to increase by nearly 50% (OR = 1.48, 95% CI: 1.06, 2.08) for each increasing step towards poorer self-reported health. A decreasing acculturation score is related (p = 0.006) to no VPA. This means that no vigorous physical activity is associated with less assimilation to the Anglo culture (OR = 0.57, 95% CI: 0.39, 0.85). We also find that an increase in body mass index is marginally associated (p = 0.062) with no vigorous physical activity with a 7% increase in odds (OR = 1.07, 95% CI: 0.99, 1.14) for every one kg/m^2 ^increase in BMI.

**Table 4:** Zero-inflated Poisson Model for Number of Days of VPA

	Logistic Portion^1^			Poisson Portion		
	Odds Ratio	95% CI	p-value	Rate Ratio	95% CI	p-value
Current employment status						
Full-time	Reference			Reference		
Part-time	0.395	0.147, 1.071	0.068	0.635	0.368, 1.098	0.10
Self-employed	0.645	0.194, 2.147	0.47	1.080	0.544, 2.143	0.83
Homemaker/other unemployed	0.603	0.265, 1.370	0.23	0.866	0.544, 1.379	0.54
Years of formal education						
Through 6^th ^grade	Reference			Reference		
Middle school	1.370	0.521, 3.597	0.52	1.331	0.748, 2.370	0.33
High school	1.649	0.609, 4.482	0.32	2.119	1.208,3.706	0.009
Any college	1.608	0.604, 4.263	0.34	2.121	1.185, 3.781	0.012
Smoked cigarettes past 30 days (Y/N)	1.465	0.563, 3.819	0.43	0.907	0.513, 1.603	0.74
Body Mass Index	1.066	0.997, 1.140	0.062	0.987	0.935, 1.042	0.63
Marital status (Married/Not married)	1.511	0.629, 3.633	0.35	1.174	0.732, 1.880	0.51
Self-reported health (1=Excellent, 5=Poor)	1.481	1.057, 2.077	0.023	0.932	0.764, 1.137	0.49
Household size	1.094	0.897, 1.335	0.38	1.040	0.953, 1.135	0.38
Age (years)	1.111	0.937, 1.323	0.23	1.089	0.994, 1.191	0.068
Acculturation score	0.574	0.388, 0.849	0.006	0.840	0.676, 1.044	0.12

^1 ^Models the probability of no vigorous physical activity

For the Poisson portion, education is related to VPA. The rate of activity is two-fold higher (RR = 2.12) when comparing either a high school education (95% CI: 1.21, 3.71) or any college (95% CI: 1.19, 3.78) to 6^th ^grade or less. In addition, age is marginally related (p = 0.068) to increasing VPA; every 5-year increase in age increases the rate by 9% (RR = 1.09, 95% CI: 0.99, 1.19).

## Conclusion

Although Poisson regression is cited as a recommended approach for analyzing count data, it often does not fit the data very well. The extra variability may be handled using modifications to the Poisson such as those described in this paper. These models may be especially useful in many epidemiological applications involving physical activity, nutrition and health services outcomes. Although analysts might consider categorizing such a skewed outcome, there are advantages to maintaining a continuous response. Software is readily available to fit these models in such packages as SAS, STATA and LIMDEP. The purpose of this article was to bring their utility to the attention of a wider audience in epidemiology.

In this example using VPA, the observed frequency of zeros was larger than expected under the Poisson regression. Our choices included inflating the standard error using a technique such as GEE, or modeling the data specifically based on an underlying framework provided by the negative binomial, ZIP or ZINB models. We found that the ZIP model yielded the best fit and may provide an interesting process in which to consider the reasons behind the preponderance of zeros.

In this study, we found that 82.4% of the subjects reported no days of physical activity. These findings are consistent with previous literature [38-40]. Evenson et al. [38] conducted face-to-face Spanish interviews with a convenience sample of 671 Latina immigrants and found that only 16.8% met VPA recommendations (≥ 20 minutes' duration for at least three days per week). We found that no VPA was associated with poorer levels of self-reported health and a decreasing acculturation score indicating less assimilation to the Anglo culture. It was marginally associated with increasing BMI. Increasing intensity of VPA was related to higher levels of education and marginally related to increasing age. Comparisons with existing findings are difficult to make given differences in physical activity outcomes. Nevertheless, some comparisons can be made.

Our findings regarding acculturation are consistent with other research indicating less physical activity with less acculturation. For example, in the NHANES study, Crespo et al [23] found that Mexican-Americans who preferred the Spanish language were less likely to report any leisure-time physical activity. Similarly, Cantero et al [41] indicated that more acculturated Latinas were more likely to exercise regularly. Results on the association between education and physical activity, and weight status and physical activity are also supported by previous research findings [17,22]. Qualitative data indicate that Latinas who exercised reported better health status, a result that is in agreement with the association observed in our study [19]. Eyler et al [39] compared correlates of physical activity among women aged 20–50 years from diverse racial/ethnic groups and found Latinas who rated their health as excellent were more likely to engage in vigorous physical activity (3–7 days/week for 20 minutes at a time) compared to those who rated their health as fair/poor. The findings on age in the current study seem to conflict with the existing literature. Most reports suggest that younger Latinas are more likely to engage in physical activity [e.g. [39]]. Overall, these data support the need for ongoing efforts to target physical activity interventions to less acculturated Latinas to prevent continued weight gain and improve well-being.

We must be cautious about accepting the ZIP model strictly on the basis of model fitting alone and, therefore, accepting as proof the idea of two subpopulations at zero [1,4]. Nevertheless, it would seem reasonable that a portion of women are not able to engage in VPA either due to a health condition or other obstacles not captured by covariates that make it impossible to participate in such activities. Whereas, there are other women reporting no VPA who could engage in such activities but choose not to, or engage in VPA but to such a limited extent that zero days are reported and represent one extreme of VPA intensity. It is on this basis that the ZIP model may be accepted and could help to describe different profiles of women.

Although the negative binomial did not provide as good a fit as the ZIP model, it was a substantial improvement over the Poisson and might be acceptable if the two-part mechanism seems inappropriate. The negative binomial identified decreasing BMI, better levels of self-reported health and an increasing acculturation score as predictive of increasing VPA. These are the same predictors identified as part of the logistic portion of the ZIP model.

Our choice of the ZIP over the ZINB model was based on parsimony since they provided a similar fit. The ZIP model does not include the random error term that allows the conditional variance of y_i _to exceed the conditional mean. But the interpretations of the model regression parameters are the same for both models.

In this example of VPA, the same set of covariates were used for both the logistic part and the intensity part of the ZIP and ZINB models. This is not necessary and one may be interested in constructing a parsimonious model for each part. However, it may be more informative and make interpretation more manageable if the same set of variables is included in each part.

Programs to extend Poisson and negative binomial regression to clustered or longitudinal data are widely available and include the SAS GENMOD procedure using GEE and the NLMIXED procedure for mixed effects regression models. Recent work has focused on extending the ZIP. For example, Yau and Lee [42] discuss a random effects ZIP model to examine an intervention to prevent injuries in a cleaning services department of a public hospital in Australia. The outcome variable was injury count collected in pre- and post-intervention periods on the same subjects. Hur, Hedeker, Henderson et al [43] describe a ZIP model with random effects to analyze the number of postoperative complications within 30 days in patients who received a partial colectomy operation. The patients (n = 3501) came from 123 Veterans Affairs Medical Centers. Thus, subjects are clustered within hospitals and this clustering must be accounted for in the analysis. Further development of software for ZIP models is underway.

## Competing interests

The author(s) declare that they have no competing interests.

## Authors' contributions

DJS conceived and designed the study, carried out the statistical analyses and drafted most of the manuscript. GXA participated in study design, developed the physical activity example, and contributed to interpreting the results. EMA participated in study design, contributed to developing the physical activity example and interpreting the results. JPE participated in study design and helped draft the manuscript. All authors read and approved the final manuscript.
